# Therapeutic potential and adverse events of everolimus for treatment of hepatocellular carcinoma – systematic review and meta-analysis

**DOI:** 10.1002/cam4.150

**Published:** 2013-10-22

**Authors:** Kenya Yamanaka, Marius Petrulionis, Shibo Lin, Chao Gao, Uwe Galli, Susanne Richter, Susanne Winkler, Philipp Houben, Daniel Schultze, Etsuro Hatano, Peter Schemmer

**Affiliations:** 1 Department of General and Transplant Surgery, University Hospital of HeidelbergHeidelberg, Germany; 2 Department of Surgery, Graduate School of Medicine, Kyoto UniversityKyoto, Japan; 3 Novartis Pharma GmbHNürnberg, Germany

**Keywords:** Adverse events, everolimus, hepatocellular carcinoma, liver transplantation

## Abstract

Everolimus is an orally administrated mammalian target of rapamycin (mTOR) inhibitor. Several large-scale randomized controlled trials (RCTs) have demonstrated the survival benefits of everolimus at the dose of 10 mg/day for solid cancers. Furthermore, mTOR-inhibitor-based immunosuppression is associated with survival benefits for patients with hepatocellular carcinoma (HCC) who have received liver transplantation. However, a low rate of tumor reduction and some adverse events have been pointed out. This review summarizes the antitumor effects and adverse events of everolimus and evaluates its possible application in advanced HCC. For the meta-analysis of adverse events, we used the RCTs for solid cancers. The odds ratios of adverse events were calculated using the Peto method. Manypreclinical studies demonstrated that everolimus had antitumor effects such as antiproliferation and antiangiogenesis. However, some differences in the effects were observed among in vivo animal studies for HCC treatment. Meanwhile, clinical studies demonstrated that the response rate of single-agent everolimus was low, though survival benefits could be expected. The meta-analysis revealed the odds ratios (95% confidence interval [CI]) of stomatitis: 5.42 [4.31–6.73], hyperglycemia: 3.22 [2.37–4.39], anemia: 3.34 [2.37–4.67], pneumonitis: 6.02 [3.95–9.16], aspartate aminotransferase levels: 2.22 [1.37–3.62], and serum alanine aminotransferase levels: 2.94 [1.72–5.02], respectively. Everolimus at the dose of 10 mg/day significantly increased the risk of the adverse events. In order to enable its application to the standard conventional therapies of HCC, further studies are required to enhance the antitumor effects and manage the adverse events of everolimus.

## Introduction

The development of radiofrequency ablation, transarterial chemoembolization (TACE), liver resection, and liver transplantation has prolonged the life expectancy of patients with hepatocellular carcinoma (HCC). However, HCC repeatedly relapses, due to intrahepatic metastases or multicentric carcinogenesis [1]. Although large-scale randomized clinical trials (RCTs) have proven that sorafenib improves the probability of survival in patients with advanced HCC [2, 3], no other molecular targeted agents and no cytotoxic agents that have survival benefits against HCC have been established. It remains a cancer with a poor prognosis.

The mammalian target of rapamycin (mTOR), which is located in the downstream of the phosphatidylinositol 3 kinase AKT pathway [4], is a key regulator of growth and proliferation of tumor cells. mTOR signaling acts through the phosphorylation of the ribosomal protein S6 kinase beta-1 (S6K1) and the eukaryotic initiation factor 4-binding protein 1 (4E-BP1) [5]. Activation of the mTOR pathway is observed in various solid cancers, including 30–40% of HCC [6–9]. mTOR-activated HCC was associated with a higher level of alpha-fetoprotein and a higher incidence of recurrence [7]. Everolimus is an mTOR inhibitor, which is designed for oral administration [10]. Everolimus binds with the intracellular receptor FK506-binding protein (FKBP-12) and forms the everolimus-FKBP12 complex to block the activation of mTOR [10]. Several large-scale RCTs have demonstrated the survival benefits of everolimus for solid cancers [11–14]. A large-scale RCT of everolimus for HCC is conducted [15].

On the other hand, everolimus has been clinically used as an immunosuppressant for patients after organ transplantation [16, 17]. It has been already administered to patients with HCC who received liver transplantation. mTOR-inhibitor-based immunosuppression is associated with survival benefits for them [18–20]. Maintenance immunosuppression with everolimus is associated with risk reduction in de novo malignancy [21]. In addition, everolimus is reportedly effective to manage patients with HCC recurrence after liver transplantation [22, 23]. Therefore, everolimus is presumed to have therapeutic potential to overcome advanced HCC.

This systematic review summarizes the antitumor effects and adverse events of everolimus demonstrated by preclinical and clinical studies to apply everolimus to standard conventional therapies of advanced HCC.

## Material and Methods

### Literature search

We manually searched the PubMed database without any restrictions for preclinical and clinical studies of mTOR inhibitors. For the meta-analysis of adverse events, we additionally used the database to select RCTs for solid cancers including the terms, everolimus and cancer. The searching was restricted to RCTs. Phase I/II trials with everolimus, subgroup analysis and meta-analysis were excluded. Information on study design, treatment regimen, study results, and adverse events were extracted from the selected literature.

### Preclinical outcome

We selected the antitumor effects of mTOR inhibitors as the preclinical outcome. These effects are considered to enhance the therapeutic potential of everolimus for clinical application.

### Clinical outcome

The clinical end points, including progression-free survival (PFS), response rate (RR), and adverse events, were extracted from the selected articles. Stomatitis, anemia, hyperglycemia, and pneumonitis were identified as typical adverse events of everolimus. In addition, we included transaminase levels, such as aspartate aminotransferase (AST) and alanine aminotransferase (ALT), to examine whether everolimus can cause liver injury. Patients with all grades of the adverse events were included in the meta-analysis.

### Statistical analysis

The odds ratio and the 95% confidence interval (CI) of patients with adverse events were calculated using the Peto method. The *I*^2^ statistics was calculated to assess the heterogeneity of the trials included. The *I*^2^ values of 0%, 25%, 50%, and 75% were estimated as no, low, moderate, and high heterogeneity, respectively [24]. The random effects model of Mantel–Maenszel and subgroup in which the RCTs with no combinative treatment were selected was used as sensitive analyses. A two-tailed *P* value of less than 0.05 was deemed statistically significant. All statistical analyses were performed using Review Manager, Version 5 (The Cochrane Collaboration, Oxford, U.K.).

## Results

### Antitumor effects of everolimus

#### Direct effects of everolimus on tumor cells

##### Antiproliferative effect

The most well-known function of mTOR is its ability to promote the synthesis of proteins involved in the cell cycle. 4E-BP1 plays a critical role in mediating tumor proliferation and progression in the mTOR pathway [25]. mTOR inhibitors decrease the action of cyclin D1/cyclin-dependent kinase (CDK)2 complex and cyclin D1/CDK4 [26, 27]. They inhibit the expression of Myc and activation of cyclin E to inhibit tumor proliferation [28]. mTOR inhibitors stop the cell cycle late in G1 to induce a G1 cell-cycle arrest [28].

The mTOR pathway integrates growth factor signals with the metabolic pathway to regulate cell growth and proliferation [29]. Tumor progression is related to Glut1 expression, which is increased by mTOR complex 1, (mTORC1) activation [30, 31]. mTOR inhibitors decrease gene expression of glucose uptake and glycolysis [29]. In addition, an increase in de novo lipid synthesis is also indispensable for tumor proliferation [32]. mTORC1 activates sterol regulatory element-binding protein (SREBP)-1 and induces lipid synthesis [33]. mTOR inhibitors reduce tumor progression and growth through SREBP-1.

##### Apoptosis

mTOR inhibitors inhibit expression of anti-apoptotic protein [34]. Rapamycin activates the c-Jun NH2-terminal kinase (JNK) pathway to induce apoptosis in absence of p53, dependent on 4E-BP1 [35], which suggests everolimus can induce apoptosis in tumors with p53 mutation [36]. Everolimus recovers the apoptotic program. Defects in the apoptotic pathway cause resistance to everolimus [34].

##### Autophagy

mTOR inhibitors are an inducer of autophagy [37]. mTOR inhibitors dephosphorylate autophagy-related gene 13 to lose its ability to bind to ULK1, thereby inducing autophagy [38]. The tumor suppressor genes, phosphatase and tensin homolog (PTEN) and p53, act on the mTOR pathway and stimulate autophagy [39, 40].

#### Indirect effects of everolimus on tumor cells

##### Antiangiogenesis

Endothelial cells are more sensitive to mTOR inhibitors than tumor cells. mTOR inhibitors act on endothelial cells to decrease the secretion of vascular endothelial growth factor (VEGF), and they obstruct VEGF-driven tubular formation, endothelial cell migration, and sprouting to control proliferation of the endothelial cell [18, 41]. Everolimus reduces Tie-2 levels and undifferentiated vessels, and it additionally controls serum and tumor VEGF [42]. It also inhibits the expression and translational activation of hipoxia inducible factor (HIF)1α to reduce VEGF production [43].

##### Thrombosis in tumor vessels

mTOR regulates the expression of tissue factor (TF) through S6K1 [44]. mTOR inhibitors increase TF of tumor endothelial cells and vascular smooth muscle cells to induce tumor-specific thrombosis. It promotes thrombosis in tumor vessels to induce tumor necrosis [45].

### Heterogeneous findings of the antitumor effects among in vivo animal studies using everolimus for HCC treatment

We found four publications regarding in vivo animal researches using everolimus for HCC treatment (Table 1) [7, 27, 37, 41]. Three of them used tumor implantation models and one study used a mouse diethylnitrosamine (DEN) tumor-induced model. The three tumor implantation models demonstrated inhibition of phosphorylation of S6K1 or 4E-BP1, but the tumor-induced model did not confirm this finding. The implantation models showed antiproliferation effect, unlike the induced model. Three of four studies showed an increase oin terminal transferase uridyl nick end labeling (TUNEL)-positive cells or upregulation of caspase 3. Among two studies that evaluated angiogenesis, inhibition of VEGF was observed in one research, while it was not observed in another study.

**Table 1:** Results of in vivo animal studies of everolimus for HCC

	Piguet etal.	Villanueva etal.	Huynh etal.	Thomas etal.
Dose, duration	5mg/kg×2/w 30days	5mg/kg×3/w 15days	2.5mg/kg/day 18days	10mg/kg 28days
Model	Tumor implantation, (Morris Hepatoma cells → ACI rats)	Tumor implantation, (Huh7 → NU/NU mice)	Tumor implantation, (4 HCC cell lines → SCID mice)	A Den-induced HCC (C57BL/6 mice treated with DEN)
mTOR activation	p4E-BP1↓, pERK→, pAKT→	pS6K1↓	pS6K1↓, p4E-BP1↓, pmTOR→, pAKT→	p4E-BP1→, pAKT↑
Necrosis	Giemsa→	N.E.	N.E.	N.E.
Apoptosis	Caspase3↑	TUNEL↑	Caspase3→	TUNEL↑
Proliferation	N.E.	Ki67↓	Ki67↓	Ki67→
Angiogenesis	VEGF→	N.E.	VEGF↓, CD31↓	N.E.
Survival benefit	+	+	N.E.	N.E.

HCC, hepatocellular carcinoma; DEN, diethylnitrosamine; N.E., not estimated; mTOR, mammalian target of rapamycin; S6K1, ribosomal protein S6 kinase beta-1; 4E-BP1, eukaryotic initiation factor 4-binding protein 1; TUNEL, terminal transferase uridyl nick end labeling; VEGF, vascular endothelial growth factor.

**Figure 1:**
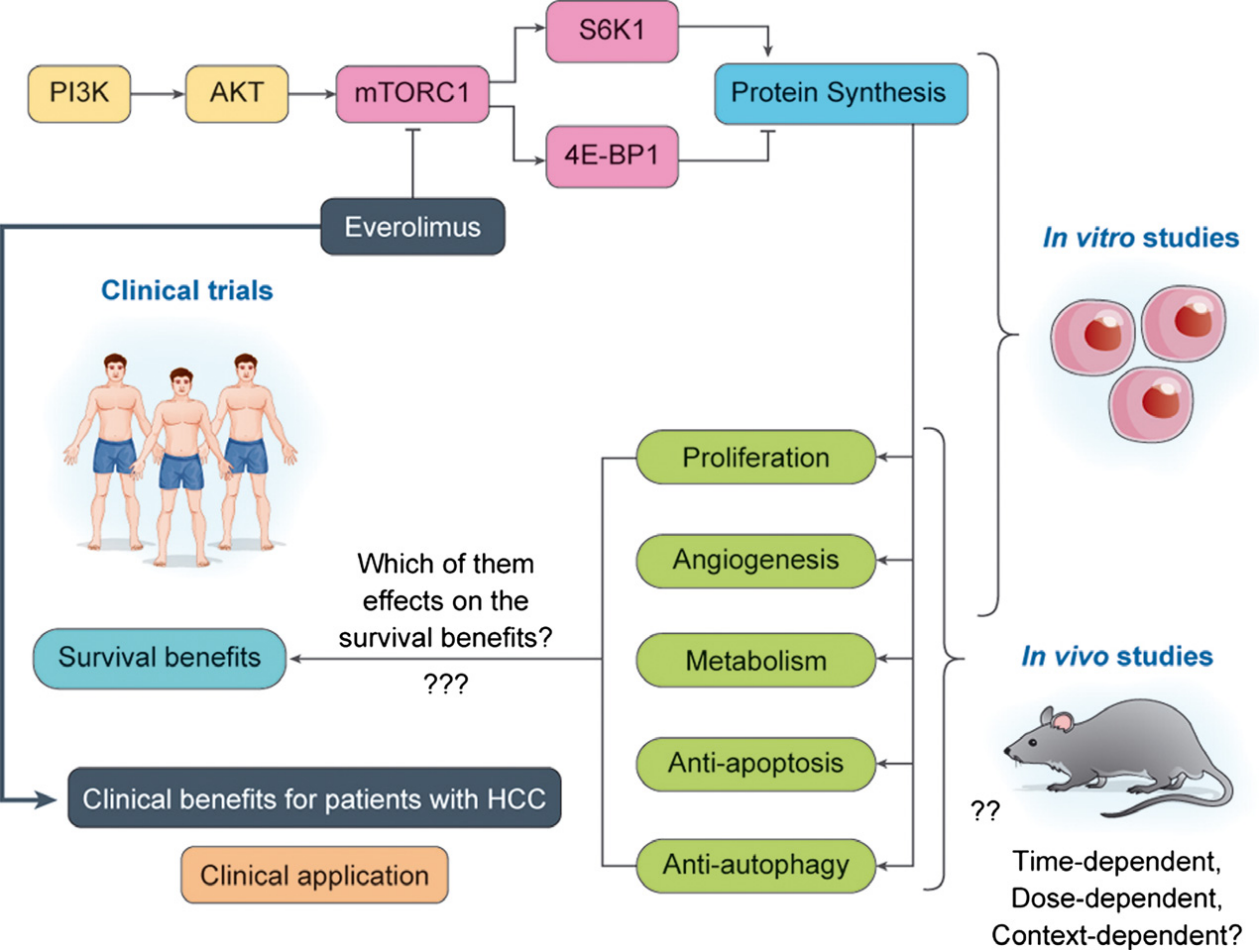
Mammalian target of rapamycin (mTOR) signal pathways and therapeutic potential of everolimus.

The effects of everolimus are considered to be time-, dose- and context-dependent [46]. There were the differences in dosage and period among animal experiments. However, heterogeneous findings existed among animal experiments, and which antitumor effects have survival benefits remained unsolved (Fig. 1).

### Effects and adverse events of everolimus

#### RR of single-agent everolimus was low, though survival benefits could be expected

From a total of 20 studies identified, we specified four published articles (Table 2) [11–14]. The targeted cancers were hormone-receptor-positive advanced breast cancer, advanced neuroendocrine tumors associated with carcinoid syndrome, advanced renal cell carcinoma, and advanced pancreatic neuroendocrine tumor. The dosage of everolimus was 10 mg/day and the primary endpoint was PFS.

**Table 2:** Characteristics of clinical trials, patients and efficacy included in the meta-analysis

Reference	Cancer	Combinative treatment	Number of patients		Median PFS (months)		HR of PFS [95% CI]	RR	
			
			Eve.	Cont.	Eve.	Cont.	Eve./Cont.	Eve.	Cont.
[11]	Postmenopausal hormone-receptor-positive advanced breast cancer	25mg exemestane/day	482	238	6.9	2.8	0.43 [0.35–0.54]	9.5%	0.4%
[13]	Advanced neuroendocrine tumors associated with carcinoid syndrome	30mg octreotide/month	216	213	16.4	11.3	0.77 [0.59–1.00]	2.3%	1.9%
[14]	Advanced RCC	No	269	135	4.0	1.9	0.30 [0.22–0.40]	1.0%	0.0%
[12]	Advanced pancreatic neuroendocrine tumor	No	207	203	11	4.6	0.35 [0.27–0.45]	5.0%	2.0%

RCC, renal cell carcinoma; PFS, progression-free survival; HR, hazard ratio; CI, confidence interval; RR, response rate; Eve, everolimus; Cont, control.

A phase I/II trial of everolimus for HCC showed that median PFS, time to progression, overall survival, and RR were 3.8, 3.9, 8.4 months and 4%, respectively [47]. Everolimus responded to the sorafenib refractory patients. Therefore, everolimus can delay tumor progression and is expected as second-line therapy in HCC resistant to sorafenib. However, RR is low after treatment of single-agent everolimus. The combination therapy with everolimus and other conventional therapies may be necessary, particularly if tumor reduction is required for the treatment of advanced HCC.

#### Everolimus increases incidence of hepatic injury in addition to adverse events such as stomatitis, anemia, hyperglycemia, and pneumonitis

**Figure 2:**
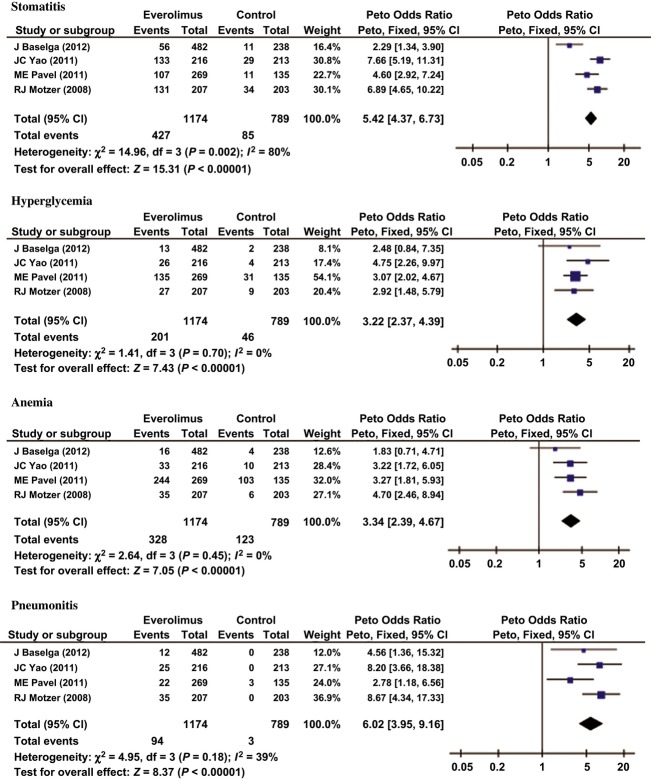
Odds ratio of everolimus-associated adverse events.

The meta-analysis for four RCTs involved 1963 patients. The odds ratios and the 95% CI of stomatitis, hyperglycemia, anemia, and pneumonitis were 5.42 [4.31–6.73] with high heterogeneity, 3.22 [2.37–4.39] with no heterogeneity, 3.34 [2.37–4.67] with no heterogeneity, and 6.02 [3.95–9.16] with moderate heterogeneity, respectively (Fig. 2). Everolimus significantly increased the incidence of these adverse events. High and significant heterogeneity was observed in stomatitis. In the random effects model of Mantel–Haenszel, which was used as a sensitivity analysis, the odds ratios and the 95% CI of stomatitis, hyperglycemia, anemia, and pneumonitis were 6.71 [3.95–11.40], 3.52 [2.36–5.25], 3.64 [2.53–5.24], and 16.97 [2.81–102.29], respectively. In the subgroup analysis using two RCTs with no combinative treatment, the odds ratios and the 95% CI of stomatitis, hyperglycemia, anemia, and pneumonitis were 7.27 [5.51–9.59], 3.87 [2.47–6.08], 3.65 [2.21–6.04], and 8.47 [5.01–14.32], respectively, and no heterogeneity was present. Everolimus also significantly increased the incidence of these adverse events in the sensitivity analyses.

**Figure 3:**
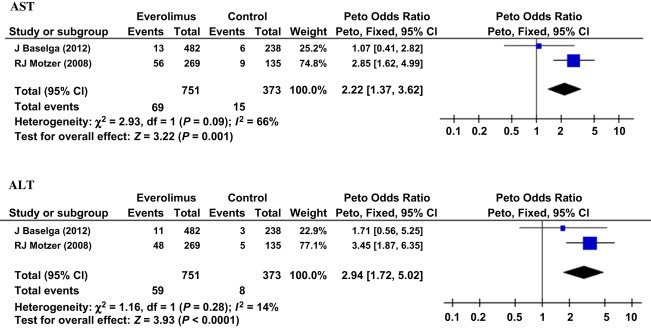
Odds ratio of everolimus-associated liver injury.

At the same time, the phase I/II trial for HCC showed the probabilities of increased levels of serum AST and ALT were observed in 36% (9/25) and 24% (6/25) and those of more than grade 3 of AST and ALT were 12% and 4%, respectively [47]. The meta-analysis using two of the four RCTs which reported serum transaminase levels reveals that the odds ratios and the 95% CI of AST and ALT were 2.22 [1.37–3.62] with high heterogeneity and 2.94 [1.72–5.02] with low heterogeneity, respectively. Everolimus significantly increased the incidence of the serum transaminase levels (Fig. 3). In the random effects model of Mantel–Haenszel, the odds ratios and the 95% CI of ALT were 3.50 [1.17–10.52], and everolimus also significantly increased the serum ALT levels. Meanwhile, the odds ratios and the 95% CI of AST were 2.07 [0.62–6.97]; no significant difference was observed. However, the odds ratios and the 95% CI of AST in the RCT with no combinative treatment was 3.68 [1.76–7.70] and everolimus was considered to increase incidence of hepatic injury.

## Discussion

This study showed that the RR of single everolimus is low, even though it would have survival benefits. Thus, some surrogate markers would be needed to evaluate the effects of everolimus in clinical setting. 18F-fluorodeoxyglucose-positron emission tomography (FDG-PET) was able to evaluate tumor glycolysis and to predict the progression of HCC [31]. FDG-PET was also suitable for the estimation of antitumor activity of everolimus [48]. FDG uptake helped to decide the optimal dosage of everolimus [49]. FDG-PET correlated AKT activation following mTOR-inhibitor therapy [50]. However, some researchers considered FDG-PET as unsuitable for evaluating the effects of everolimus, as everolimus prevents glucose metabolism by using a mechanism independent of its antitumor effects [46]. Meanwhile, S6K1 inhibition in peripheral blood mononuclear cells (PBMCs) was reportedly correlated with inhibition in tumor tissues in preclinical models [51]. Inhibition of the mTOR pathway in skin was also closely associated with inhibition in tumor [52].

Combination therapy for advanced HCC is highly expected to enhance the antitumor effects of everolimus. Everolimus displays synergic effects with several cytotoxic agents and enhances chemosensitivity in HCC [53, 54]. Furthermore, everolimus enhances cisplatin-induced apoptosis by reducing cellular levers of p21 [55]. A phase I study of everolimus plus low-dose cisplatin demonstrated that the adverse events were similar to those of everolimus monotherapy [56]. Hepatic arterial infusion chemotherapy (HAIC) is performed for patients with HCC with vascular invasion, and cisplatin is administered as a standard agent in HAIC [57, 58]. TACE is a standard therapy for patients with intermediate stage HCC [1]. Cisplatin may be more effective than epirubicin in TACE for multiple HCC [59]. Therefore, the combination therapy with TACE or HAIC is expected to have some beneficial effects. Furthermore, TACE-refractory HCC is often observed after repeated TACE treatments [60, 61]. Ineffective TACE is considered to induce a neoangiogenic reaction that leads to HCC regrowth [62]. The antiangiogenic effects of everolimus may inhibit the neoangiogenic reaction to overcome TACE-refractory HCC.

Radiation therapy in HCC has an effect on tumor thrombus [63, 64]. mTOR inhibitors enhanced radiation damage of tumor vasculature [65]. Everolimus also controls the production of VEGF to increase the radiosensitivity of tumors [66]. The benefits of combination therapy with everolimus and radiation therapy are expected for the treatment of advanced HCC.

This study also demonstrated that administration of everolimus at the dosage of 10 mg/day increased the incident of liver injury in addition to the typical adverse events. Patients with worse liver function have a higher blood concentration level of everolimus [51, 67]. Although the phase I/II trial of everolimus for HCC recommended that the dosage should be 10 mg/day [47], another phase I clinical study reported that the maximum tolerated dose of everolimus in HCC was 7.5 mg/day [68]. The ongoing phase III trial for HCC decreases the dose of everolimus from 10 to 7.5 mg/day. On the other hand, the dosage of everolimus as an immunosuppressive agent is 2.5 mg/day. Even after the dose reduction to 7.5 mg/day, a higher dose of everolimus is administered to patients with HCC, rather than for organ-transplanted patients. In liver transplantation, an average dosage of 1.3–2.9 mg/day (maximum dosage: 4 mg/day) of everolimus was administered to patients with HCC recurrence [22, 69, 70]. In case of the 10 mg/day dosing for cancer treatments, the trough level of everolimus was reported to be 13.2 ng/mL (13.8 nmol/L) and the maximum concentration was 61 ng/mL (63.7 nmol/L) [51]. In PROTECT study to evaluate nephroprotective effects of everolimus as an immunosuppressive agent, the targeted trough level was adjusted to be 5–12 ng/mL (5.23–12.5 nmol/L) and everolimus was administered at a mean dose of 4.4 mg/day [16]. However, preclinical studies have not demonstrated the relationship between the dosage of everolimus and the antitumor effects, and which antitumor effects have survival benefits remains unsolved. The correlation of serum everolimus levels and occurrence of adverse events has not been clarified, either. In addition, mTOR inhibitors enhance hepatitis B virus (HBV) replication, though it is not clarified whether mTOR inhibitor suppresses hepatitis C virus (HCV) replication in patients with HCC [71, 72]. There are several case reports of death due to reactivation of HBV by everolimus. Therefore, we will need further studies to apply the expected antitumor effects to clinical practices in HCC as early as possible.

In conclusion, everolimus, an mTOR inhibitor, is a molecular-targeted agent that has the potential to treat advanced HCC. However, heterogeneous findings of the antitumor effects have been observed among animal studies for HCC treatment. RR of single-agent everolimus was low, and it increases incidence of liver injury in addition to stomatitis, anemia, hyperglycemia, and pneumonitis. To improve the prognosis of advanced HCC, further studies are required to both enhance the antitumor effects as well as manage the adverse events of everolimus.
